# Indoor Solid Fuel Use and Non-Neoplastic Digestive System Diseases: A Population-Based Cohort Study Among Chinese Middle-Aged and Older Population

**DOI:** 10.3389/ijph.2022.1605419

**Published:** 2022-12-21

**Authors:** Yahang Liu, Silu Zeng, Chen Huang, Ce Wang, Jingjing Zhu, Jiahuan Peng, Fengfei Ding, Jiong Li, Guoyou Qin, Jiaohua Chen

**Affiliations:** ^1^ Department of Biostatistics, School of Public Health, The Key Laboratory of Public Health Safety of Ministry of Education, Fudan University, Shanghai, China; ^2^ Clinical Research Unit, Shanghai Ninth People’s Hospital Affiliated to Shanghai Jiaotong University School of Medicine, Shanghai, China; ^3^ Department of Pharmacology, School of Basic Medical Sciences, Fudan University, Shanghai, China; ^4^ Department of Clinical Medicine, Aarhus University, Aarhus, Denmark; ^5^ Department of Clinical Epidemiology, Aarhus University, Aarhus, Denmark; ^6^ Department of Health Management, Seventh People’s Hospital of Shanghai University of Traditional Chinese Medicine, Shanghai, China

**Keywords:** solid fuels, household air pollution, clean fuels, digestive diseases, middle-aged and older adults

## Abstract

**Objectives:** We tended to explore the association of indoor air pollution (IAP) and non-neoplastic digestive system diseases (NNDSD) among the Chinese middle-aged and older population.

**Methods:** From 2011 to 2018, we included 7884 NNDSD-free adults from the China Health and Retirement Longitudinal Study (CHARLS). Physician-diagnosed NNDSD was obtained by self-reported information at baseline and updated across follow-up surveys. We investigated the associations between baseline exposure of solid fuel use for cooking and/or heating and NNDSD diagnosed during follow-up through Cox proportional hazard models. Furthermore, we examined the relationship between cooking fuel switching and NNDSD diagnosed during follow-up.

**Results:** Solid fuel use for cooking and/or heating was positively associated with NNDSD after adjusting for potential confounders. The risk of NNDSD among subjects who always use solid fuel for cooking (adjusted hazard ratio [aHR]: 1.42; 95% confidence interval [CI]: 1.09, 1.84) was higher than those with always clean fuels. Moreover, we found a lower NNDSD risk among participants who switched from solid to clean cooking fuel (aHR: 0.65; 95% CI: 0.49, 0.87) than those with always solid fuels.

**Conclusion:** Our present study shows that indoor solid fuel use is a dependent risk factor for NNDSD. Moreover, switching to clean fuel may contribute to the prevention of digestive system illnesses.

## Introduction

Exposure to indoor air pollution (IAP) ranks the top ten leading risks for diseases worldwide [1, 2]. Approximately 2.5 billion people worldwide are exposed to IAP from cooking with solid fuels, and the number increases when including those heating and lighting with solid fuels [3]. Incomplete combustion of solid fuels in developing countries becomes a major source of exposure to IAP (4). Solid fuel-related pollutants mainly include polycyclic aromatic hydrocarbons, particulate matter, nitrous oxide, carbon monoxide, and sulfur dioxide, which are two to three-fold higher in indoor environments than outdoors. The use of solid fuels is a particularly pressing issue in China [4]. Although the proportion of people exposed to indoor air pollution from solid fuels declines, 32% of the Chinese population still use solid fuels for cooking or heating [4]. It is estimated that 271,089 (209,882 to 346,561) deaths in China were attributable to solid fuel use in 2017(3).

Digestive tract disorders have become global diseases with accelerating increased incidence in countries whose societies have become westernized, like China [5, 6]. Moreover, evidence has shown that some non-neoplastic digestive system diseases (NNDSD), such as helicobacter pylori infection and chronic gastritis, are precursors to the development of digestive cancers [7–9]. Identifying potential risk factors for NNDSD may help prevent the development of NNDSD and gastrointestinal tumors. Previous epidemiological investigations have reported that exposure to ambient pollutants, such as ozone, contributes to a higher risk of digestive diseases [10–14]. However, the effect of IAP caused by solid fuel on NNDSD is incompletely understood with an epidemiological gap. Moreover, past studies on the relationship between air pollution and digestive diseases mainly focused on short-term exposure to specific pollutants [11, 13].

Hence, to partly provide epidemiological evidence for this topic, we assessed whether long-term chronic exposure to cooking and heating solid fuel use separately or simultaneously is associated with non-neoplastic digestive system diseases. We also explored whether the risk of NNDSD in participants who have switched from solid to clean fuel was lower compared to always using solid fuel.

## Methods

### Study Population

The current study was based on the China Health and Retirement Longitudinal Study (CHARLS), aiming to collect high-quality data representative of middle-aged and elderly people in China [15]. All participants underwent an interview to collect data on sociodemographic characteristics, health status, and medical history. The CHARLS baseline survey was conducted in 2011, with regular follow-up visits every 2–3 years in 2013, 2015, and 2018, respectively. All participants provided written informed consent during the investigation. A detailed description is available on its website (http://charls.pku.edu.cn/en).

**FIGURE 1:**
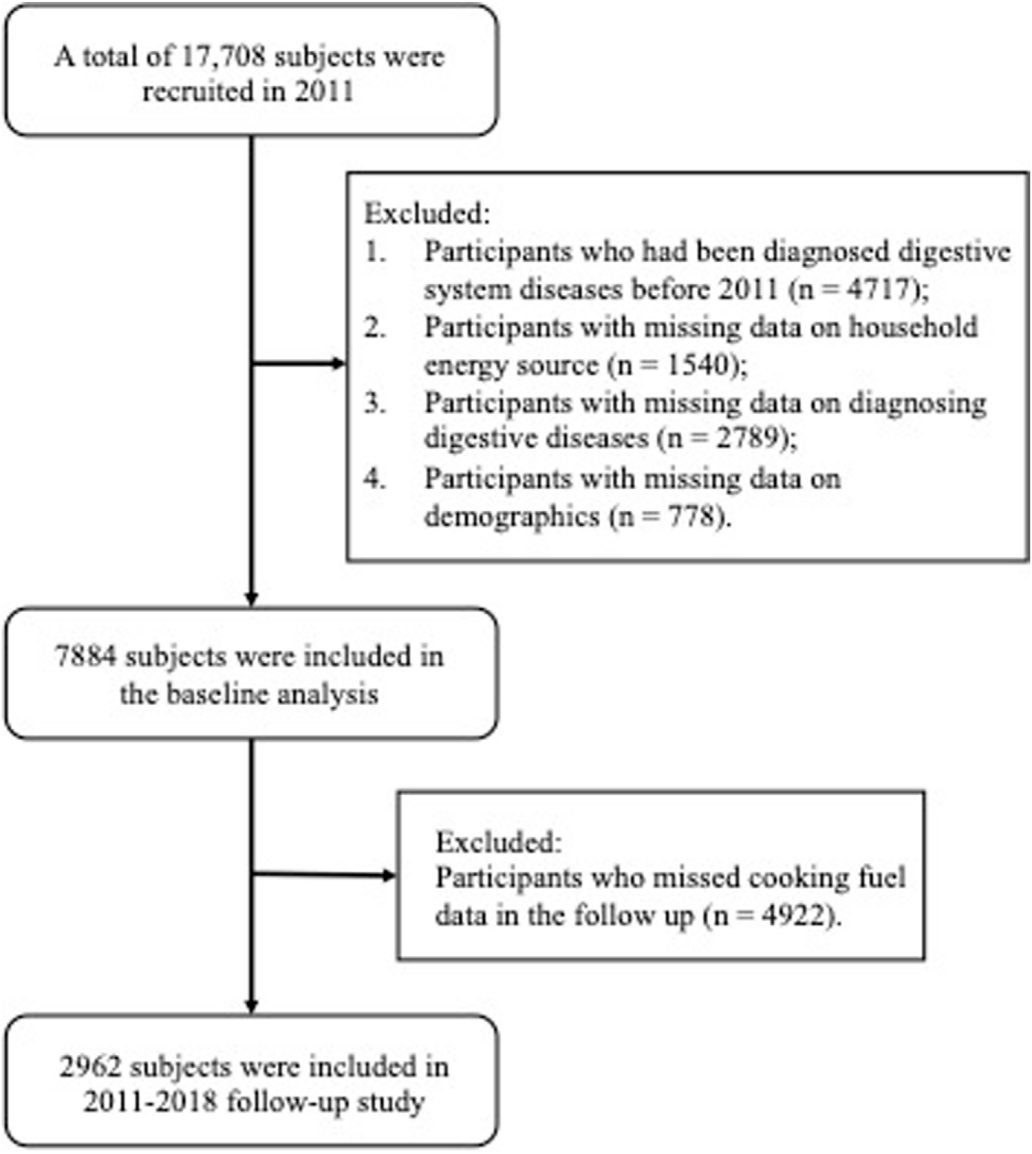
Flowchart of the selection process of population (China Health and Retirement Longitudinal Study, China, 2011, 2013, 2015, 2018).

17,708 adults were recruited in 2011. We excluded participants who had been diagnosed of non-neoplastic digestive system diseases (NNDSD) in 2011 and before (*n* = 4717), participants with missing data on indoor fuel source (*n* = 1540), diagnosing NNDSD (*n* = 2789) and demographic characteristics (*n* = 778); then 7,884 participants were left for the baseline analysis of the use of cooking and heating fuel types and their combined effects on NNDSD. For the follow-up analysis, we additionally excluded 4922 participants with missing data on cooking fuel type, resulting in 2962 individuals for analysis (Figure 1). Since data on heating fuel in 2015 were not available, we analyzed the effect of switching fuel types on the risk of NNDSD only regarding cooking fuel data.

### Outcome of Interest

Physician-diagnosed NNDSD after 2011 was defined as the outcome of interest. Physician-diagnosed NNDSD was obtained from a self-reported questionnaire across follow-up surveys (“Have you been diagnosed with stomach or other non-neoplastic digestive system diseases by a doctor?”). If the subject’s answer to this question was “Yes”, he or she was defined as suffering from NNDSD. The survival time was defined as the time from the baseline date to the dates of the NNDSD diagnosis, loss to follow-up, or the end of follow-up (2018), whichever occurred first.

### Indoor Energy Source

We dichotomized cooking fuels and heating fuels [clean fuels (natural gas, marsh gas, liquefied petroleum gas, solar, electric, or concentration heating) vs. solid fuels (coal, crop residues, wood-burning, or others)]. Notably, heating fuel information use was not available in 2015–2016. Therefore, we evaluated the effect of fuel type conversion on NNDSD only with the cooking fuel data in the follow-up analysis.

We divided the population into four groups in the follow-up analysis based on the type of fuel used at baseline and updated across follow-up visits: always solid fuel, always clean fuel, solid-to-clean fuel, and clean-to-solid fuel. The NNDSD emerged after fuel type conversion. For example, a subject who was diagnosed with NNDSD firstly in 2013, used solid energy in 2011 and clean energy in 2013, would be classified into the solid-to-clean fuel group; a subject who was a clean fuel user in 2011 while solid fuel user in 2015, diagnosed with NNDSD firstly in 2015, would be divided into clean-to-solid fuel group. Other groups were defined similarly.

### Covariates

Covariates adjusted in our analyses included sex, age (middle-aged adults, 45–65 years; old adults, >65 years), educational level (illiteracy or informal education; elementary school or above), marital status (married, unmarried), smoking status (smokers, non-smokers), residence region (city or town; village), self-reported economic level (relatively poor or poor, average, relatively high or high), hypertension comorbidity (yes, no), diabetes (yes, no), dyslipidemia (yes, no), cardiovascular disease (yes, no), stroke (yes, no), liver diseases (yes, no), lung diseases (yes, no), cancer or malignant tumor (yes, no), and kidney diseases (yes, no).

The educational level was obtained by question, “what’s the highest level of education you have attained now?” Then, the answers were classified into two groups (illiteracy or informal education, elementary school or above). Marital status was divided into married and unmarried. Non-smokers were defined as those who had never smoked, and smokers were defined as those who had smoked or were currently smoking. Systolic blood pressure (SBP) or diastolic blood pressure (DBP) over 140/90 mmHg was defined as hypertension. Diabetes was defined as [1]: had been diagnosed as diabetes by a clinical doctor [2], fasting blood glucose (FBG) >200 mg/dl (11.1 mmol/L). HDL-C, LDL-C, glucose, and triglyceride were obtained from blood examination. Cardiovascular disease, stroke, liver diseases, lung diseases, cancer or malignant tumors, and kidney diseases were defined as previously diagnosed with this disease by a clinical doctor. Cardiovascular diseases included heart attack, coronary heart disease, angina, congestive heart failure, and other problems.

### Statistical Analyses

Descriptive statistical analysis was used to compare population characteristics according to the indoor fuel types at baseline and fuel type change patterns during follow-up. The categorical variables were presented as frequency (proportions).

We performed Cox proportional hazards regression models to calculate adjusted hazard ratios (aHRs) with 95% confidence intervals (CIs) for the association between indoor solid fuel and NNDSD. We evaluated the proportional hazards assumption using the accumulated martingale residuals and found no obvious violation of the proportional hazards assumption [16]. In the baseline analysis, we estimate the relationship between cooking or heating fuel use, separately or simultaneously, and the risk of NNDSD among 7,884 participants. In the follow-up analysis, we evaluated the association between cooking fuel type conversion and the risk of NNDSD among 2962 participants. We conducted stratified analyses by baseline characteristics including age, sex, hypertension, and smoking status with the fully adjusted model.

Three models were fitted: Model 1 was a crude model; Model 2 adjusted for age, sex, and educational level; Model 3 was a fully adjusted model with additional adjustment for marital status, smoking status, residence region, self-reported economic level, hypertension comorbidity, cardiovascular disease, stroke, liver diseases, lung diseases, cancer or malignant tumor and kidney diseases.

We performed sensitivity analyses based on our primary Cox models. Firstly, we analyzed the association of household fuels conversion and NNDSD among non-smokers. Secondly, we performed stratified analyses by baseline characteristics to assess whether they could be effect modifiers of the studied association, including age, gender, smoking, and hypertension. Thirdly, we also performed sub-analyses with additional adjustment for population weighting concentrations of PM2.5 ( $\mu g/m^{3}$ ) and geographical weighting concentrations of PM2.5. Fourthly, we examined the associations between household fuel exposure at baseline and NNDSD stratified by comorbidities using Cox models.

All analyses were performed by SAS version 9.4 (SAS Institute, Cary, NC, United States), and R 4.1.1. A two-sided test *p* < 0.05 was considered statistically significant.

## Results

### Basic Characteristics of the Study Population

The baseline characteristics of the participants in the baseline and follow-up analyses are shown according to the indoor fuel patterns (Table 1). We included 7,884 and 2962 enrolled participants in the baseline and follow-up analyses. Solid fuel was used for cooking by 3,709 (47.04%) participants and for heating by 3,732 (47.34%) participants. Solid cooking and heating fuel users were more inclined to be older, have families in poor financial conditions, be more likely to be smokers, and be illiterate or informally educated. 803 (27.11%) participants always used solid fuel during the follow-up period, 1,352 (45.6%) always used clean fuel, and 176 (5.94%) used fuel types that have switched from clean to solid fuel. Compared with participants who always used solid fuel, subjects who always used clean fuel and changed from solid to clean fuel tended to be females, aged from 45 to 65 years old, unmarried, and have a relatively high education level and high economic level at baseline.

**TABLE 1:** Summary of participants’ characteristics in baseline (2011) and follow-up study (2011–2018) (China Health and Retirement Longitudinal Study, China, 2011, 2013, 2015, 2018).

Characteristics	Household fuel use at baseline						Cooking fuel changes during follow up				
	Cooking			Heating			Always clean	Solid to clean	Clean to solid	Always solid	p
	Clean	Solid	p	Clean	Solid	p					
N	4175(53.0)	3709(47.0)		4152(52.7)	3732(47.3)		1,352(45.6)	631(21.3)	176(5.9)	803(27.1)	
Sex			0.982			0.982					0.194
Male	2034(48.7)	1808(48.8)		2024(48.8)	1818(48.7)		634(46.9)	279(44.22)	83(47.2)	401(49.9)	
Female	2141(51.3)	1901(51.2)		2128(51.2)	1914(51.3)		718(53.1)	352(55.8)	93(52.8)	402(50.1)	
Age			<0.001			<0.001					<0.001
45–65	3375(80.8)	2716(73.2)		3338(80.4)	2753(73.8)		992(73.4)	410(65.0)	122(69.3)	537(66.9)	
>65	800(19.2)	993(26.8)		814(19.6)	979(26.2)		360(26.6)	221(35.0)	54(30.7)	266(33.1)	
Region			<0.001			<0.001					<0.001
City/town	3669(87.9)	3662(98.7)		3701(89.1)	3630(97.3)		1,182(87.4)	615(97.5)	170(96.6)	796(99.1)	
Village	506(12.1)	47(1.3)		451(10.9)	102(2.7)		170(12.6)	16(2.5)	6(3.4)	7(0.9)	
Smoking			<0.001			0.061					0.010
Smokers	1,544(37.0)	1,522(41.0)		1,574(37.9)	1,492(40.0)		538(39.8)	276(43.7)	65(36.9)	372(46.3)	
Non-smokers	2631(63.0)	2187(59.0)		2578(62.1)	2240(60.0)		814(60.2)	355(56.3)	111(63.1)	431(53.7)	
Marital status			0.535			0.014					0.399
Married	3720(89.1)	3321(89.5)		3742(90.1)	3299(88.4)		1,027(76.0)	481(76.2)	140(79.6)	632(78.7)	
Unmarried	455(10.9)	388(10.5)		410(9.9)	433(11.6)		325(24.0)	150(23.8)	36(20.4)	171(21.3)	
Economy standard			<0.001			<0.001					<0.001
Poor	1,600(38.3)	1751(47.2)		1,627(39.2)	1724(46.2)		539(39.8)	301(47.7)	74(42.1)	390(48.6)	
Average	2435(58.3)	1874(50.5)		2395(57.7)	1914(51.3)		752(55.7)	309(50.0)	99(56.2)	399(46.7)	
High	140(3.4)	84(2.3)		130(3.1)	94(2.5)		61(4.5)	21(3.3)	3(1.7)	14(1.7)	
Education			<0.001			<0.001					<0.001
Illiteracy or informal education	1,483(35.5)	2052(55.3)		1,533(36.9)	2002(53.6)		488(36.1)	344(54.5)	87(49.4)	504(62.8)	
Elementary school or above	2692(64.5)	1,657(44.6)		2619(63.1)	1730(46.4)		864(63.9)	287(45.5)	89(50.6)	299(37.2)	
Hypertension			0.060			<0.001					0.312
Yes	955(22.9)	783(21.1)		990(23.8)	748(20.0)		334(24.7)	146(23.1)	42(23.9)	170(21.2)	
No	3220(77.1)	2926(78.9)		3162(76.2)	2984(80.0)		1,018(75.3)	485(76.9)	134(76.1)	633(78.8)	
Liver disease			0.631			0.891					0.200
Yes	119(2.9)	99(2.7)		116(2.8)	102(2.7)		42(3.1)	21(3.3)	1(0.6)	21(2.6)	
No	4056(97.1)	3610(97.3)		4036(97.2)	3630(97.3)		1,310(96.9)	610(96.7)	175(99.4)	782(97.4)	
Lung disease			0.001			0.019					0.415
Yes	292(7.0)	333(9.0)		301(7.3)	324(8.7)		107(7.9)	57(9.0)	14(8.0)	80(10.0)	
No	3883(93.0)	3376(91.0)		3851(92.7)	3408(91.3)		1,245(92.1)	574(91.0)	162(92.0)	723(90.0)	
Cancer or malignant tumor			0.133			0.209					0.707
Yes	40(1.0)	24(0.7)		39(0.9)	25(0.7)		10(0.7)	4(0.6)	2(1.1)	4(0.5)	
No	4135(99.0)	3685(99.3)		4113(99.1)	3707(99.3)		1,342(99.3)	627(99.4)	174(98.9)	799(99.5)	
Heart disease			0.684			<0.001					0.273
Yes	355(8.5)	305(8.2)		401(9.7)	259(6.9)		128(9.5)	53(8.4)	9(5.1)	71(8.8)	
No	3820(91.5)	3404(91.8)		3751(90.3)	3473(93.1)		1,224(90.5)	578(91.6)	167(94.9)	732(91.2)	
Stroke			0.867			0.276					0.027
Yes	78(1.89)	67(1.8)		83(2.0)	62(1.7)		30(2.2)	21(3.3)	2(1.1)	9(1.1)	
No	4097(98.1)	3642(98.2)		4069(98.0)	3670(98.3)		1,322(97.8)	610(96.7)	174(98.9)	794(98.9)	
Kidney disease			0.156			0.066					0.408
Yes	188(4.5)	193(5.2)		183(4.4)	198(5.3)		60(4.4)	31(4.9)	11(6.3)	47(5.9)	
No	3987(95.5)	3516(94.8)		3969(95.6)	3534(94.7)		1,292(95.6)	600(95.1)	165(93.7)	756(94.1)	

Note: N (%) was used for categorical variables: categorical variables were compared using chi-square tests analysis.

### The Effects of Indoor Fuel Types on Non-Neoplastic Digestive Diseases at Baseline

In the fully adjusted models, compared to individuals who used clean fuel in the baseline, solid cooking fuel users showed a significantly increased risk of non-neoplastic digestive system diseases (NNDSD) (adjusted Hazard Ratio [aHR]: 1.17; 95% confidence interval [95% CI]: 1.02, 1.34). Similar results were found between solid (vs. clean) heating fuel use and NNDSD (aHR: 1.21; 95% CI: 1.07, 1.38). In addition, compared with those who were using clean fuel for both cooking and heating, the risk of NNDSD was found to be higher only in participants both cooking and heating with solid fuel (aHR: 1.34; 95% CI: 1.15, 1.55) (Table 2).

**TABLE 2:** The associations between baseline household fuel exposure and NNDSD diagnosed during follow-up using Cox models (China Health and Retirement Longitudinal Study, China, 2011, 2013, 2015, 2018).

Variables	Events (rate/per 1,000 person years)		aHR (95%CI)
Cooking fuel			
Clean	510(18.62)	Reference	
Solid	551(22.64)	Model 1	1.23(1.09, 1.39)
		Model 2	1.21(1.07, 1.37)
		Model 3	1.18(1.02, 1.33)
Heating fuel			
Clean	510(18.53)	Reference	
Solid	551(22.77)	Model 1	1.22(1.08, 1.37)
		Model 2	1.20(1.06, 1.35)
		Model 3	1.18(1.03, 1.37)
Cooking and heating fuel			
Both clean for cooking and heating	362(17.97)	Reference	
Clean for cooking and solid for heating	148(20.04)	Model 1	1.12(0.92, 1.35)
		Model 2	1.11(0.91, 1.34)
		Model 3	1.10(0.92, 1.35)
Clean for heating and solid for cooking	148(20.44)	Model 1	1.14(0.94, 1.38)
		Model 2	1.12(0.92, 1.36)
		Model 3	1.11(0.90, 1.39)
Both solid for cooking and heating	403(23.77)	Model 1	1.33(1.15, 1.53)
		Model 2	1.30(1.12, 1.50)
		Model 3	1.31(1.12, 1.53)

Model 1 is a crude model; Model 2 adjusts for age, sex, and educational level; Model 3 adjusts for age, sex, educational level, marital status, residence region, and smoking status. Rate: incidence rate per 1,000 person-years of follow-up, equal to (number of NNDSD events)/(person-years) × 1000.

### The Association of Cooking Fuel Type Conversion With Non-Neoplastic Digestive Diseases

In the fully adjusted model of the follow-up analysis, we found that those who with always solid cooking fuel had a higher risk (aHR: 1.42; 95% CI: 1.09, 1.84) of NNDSD compared to those with always clean cooking fuel; and those who switched from solid fuel to clean fuel had a lower risk (aHR: 0.65; 95% CI: 0.49, 0.87) of NNDSD compared to those with always solid cooking fuel (Table 3).

**TABLE 3:** The associations between fuel change patterns and NNDSD during follow-up using Cox models (China Health and Retirement Longitudinal Study, China, 2011, 2013, 2015, 2018).

Variables	Events(rate)		aHR (95%CI)
Consistent fuel type			
Always clean fuel	159(17.81)	Reference	
Always solid fuel	137(26.56)	Model 1	1.50 (1.19, 1.88)
		Model 2	1.47 (1.16, 1.86)
		Model 3	1.42 (1.09, 1.84)
Had switched fuel type			
Always solid fuel	137(26.56)	Reference	
Solid to clean fuel	73(17.22)	Model 1	0.65 (0.49, 0.86)
		Model 2	0.65 (0.49, 0.87)
		Model 3	0.65 (0.49, 0.87)

Model 1 is a crude model; Model 2 adjusts for age, sex, and educational level; Model 3 adjusts for age, sex, educational level, marital status, residence region, and smoking status. Rate: incidence rate per 1,000 person-years of follow-up, equal to (number of NNDSD events)/(person-years) × 1000.

### Sensitivity Analyses

Males (aHR: 1.66; 95% CI: 1.16, 2.35), over 65 years participants (aHR: 2.63; 95% CI: 1.65, 4.21) and people without hypertension (aHR: 1.48; 95% CI: 1.12, 1.96) who consistently used solid fuels for cooking had a higher risk of NNDSD than those who continued to use clean fuels for cooking. Compared with those who consistently used solid fuels for cooking, participants aged 65+ (aHR: 0.44, 95% CI: 0.26, 0.73), those without hypertension (aHR: 0.65, 95% CI: 0.47, 0.90), men (aHR: 0.65, 95% CI: 0.43, 0.99) and women (aHR: 0.63, 95% CI: 0.42, 0.94) switching cooking fuels from solid to clean had significantly lower risk of NNDSD (Table 4).

**TABLE 4:** The subgroup analyses the association between cooking fuel use and NNDSD from 2011 to 2018 (China Health and Retirement Longitudinal Study, China, 2011, 2013, 2015, 2018).

Subgroups	Events (rate)	aHR(95%CI)
(A)		
Always solid cooking fuel		
Age(years)		
45–65	86 (24.61)	1.18 (0.88, 1.57)
>65	51 (30.65)	2.63 (1.65, 4.21)
Gender		
Male	71 (27.62)	1.66 (1.16, 2.35)
Female	66 (25.51)	1.30 (0.93, 1.82)
Hypertension		
Yes	31 (28.65)	1.52 (0.93, 2.49)
No	106 (26.01)	1.48 (1.12, 1.96)
(B)		
Solid to clean cooking fuel		
Age(years)		
45–65	49 (17.79)	0.74 (0.52, 1.05)
>65	24 (16.16)	0.44 (0.26, 0.73)
Gender		
Male	35 (18.60)	0.65 (0.43, 0.99)
Female	38 (16.12)	0.63 (0.42, 0.94)
Hypertension		
Yes	18 (18.35)	0.59 (0.32, 1.09)
No	14 (4.30)	0.65 (0.47, 0.90)

The multivariable-adjusted model adjusts for age, sex, educational level, marital status, residence region, and smoking status. Rate: incidence rate per 1,000 person-years of follow-up, equal to (number of NNDSD events)/(person-years) *1000. (A) The reference group is consistently clean cooking fuel. (B) The reference group is always solid cooking fuel.

The subgroup analyses stratified by baseline characteristics showed that males (aHR: 1.50; 95%CI: 1.21, 1.87), people aged 45–65 years (aHR: 1.37; 95%CI: 1.16, 1.63), and subjects without hypertension history (aHR: 1.35; 95%CI: 1.14, 1.60) using solid fuel for both cooking and heating had a significantly greater risk of NNDSD than those with clean cooking and heating fuels (Supplementary Figure S2). Similar results to the primary analyses were observed in subgroup analyses stratified by comorbidities, restricted to non-smokers, and additionally adjusting for population weighting concentrations of PM2.5 ( $\mu g/m^{3}$ ) and geographical weighting concentrations of PM2.5 (Supplementary Tables S2, S3, S5).

## Discussion

Using the data from a large population-based cohort study, we observed that exposure to indoor air pollution (IAP) for heating and/or cooking was positively associated with non-neoplastic digestive system diseases (NNDSD) in both the baseline and the follow-up analyses. In addition, after switching fuel types from solid to clean, the risk of NNDSD was significantly lower than those with always solid fuel.

Several epidemiological types of research have reported evidence across countries and populations on the relationship between IAP and a wide range of adverse health events, including cardiovascular events [17], cardiopulmonary mortality [18], active tuberculosis [19], cervical cancer [20], incident arthritis [21], cognitive impairment [22]. Besides, limited empirical evidence on the relationship of air pollution with digestive diseases has been proposed [10, 12, 13]. An elderly population-based study from Hong Kong showed that short-term elevations in ambient nitrogen dioxide may increase the risk of bleeding peptic ulcers and consequent emergency hospital admissions [13]. An Italian study found that air pollution was associated with increased emergency room visits of gastroenteric disorder in children aged 0–2 years [10]. A multicity case-crossover study suggested that exposure to O_3_ frequently may increase the risk of perforated appendicitis [12]. Empirical evidence on the association of air pollution with the digestive disease remains preliminary, as the type of study design, short-term exposure to pollutants, or limitations in the study population that do not allow long-term follow-up analyses of exposure and outcomes; besides, the case-crossover study might be prone to information bias [10, 12]. However, less is known about the relationship between IAP (mainly caused by indoor solid fuel use) and digestive system diseases. The present large population-based cohort study first revealed an increased risk of NNDSD in individuals with long-term cooking and/or heating solid energy. Moreover, we also evaluated the effect of IAP change on NNDSD, which has rarely been considered in previous studies [10, 12, 13]. Our results partly provide evidence of the association between IAP and NNDSD.

A Longitudinal Healthy Longevity Survey in rural China reported that switching cooking fuel types crucially influenced the health effects and that switching from solid to clean fuel for cooking showed a significantly lower risk of decreased kidney function compared to consistently use of solid cooking fuel [23]. In the present study, we found that individuals who switched from solid to clean fuels for cooking had a significantly lower risk of NNDSD than those who continued to use solid fuels for cooking. More specifically, those aged more than 65 years, non-smokers, and without hypertension comorbidity had a substantially lower risk of NNDSD. This transformation in the type of cooking fuel is in line with the government’s three-year action plan (2013–2015) to address electricity use in non-electrified areas, which will promote the use of clean energy in part [24]. Cooking fuel type conversion is a modifiable behavior suggesting that increased financial support may promote clean fuel use and digestive disease prevention.

Several hypotheses underlying the association of air pollution and digestive system disorders have been proposed [1]: The pollutants can impair digestive system function, i.e., increasing intestinal permeability, altering the gut microbiome, and even increasing the risk of digestive organ cancers [13, 25, 26]. [2] Inhalation of higher concentrations of particulate matter can alter the metabolic level and stress hormones [27]. [3] Accumulating the detrimental effect of inhaled pollutants on lung inflammation and oxidative stress may contribute to systemic inflammation and oxidative stress [28, 29]. An animal study found significantly elevated mRNA expression of TNF-*α* in colon samples of mice exposed to particulate matter, suggesting that changes in the microbiota may induce gastrointestinal inflammation [24]. [30] The specific ambient pollutants may directly reach and interact with digestive organs [31, 32]. Overall, there is no systematic mechanism explaining the disturbance of the digestive system caused by solid fuel combustion, and further research is needed.

Previous studies about the impact of air pollution on sex have yielded heterogeneous results [33–35]. Most investigations have revealed that women are more likely to be damaged by air pollution, but some studies have reported the opposite [33–35]. In the baseline analysis, we observed a more pronounced effect of solid fuel on NNDSD in males than females. Smoking is widely recognized as another major cause of IAP(36), which produces many air pollutants, some of which are the same as those produced by solid fuel combustion and are hazardous to many human systems [36]. Published studies have documented smoking was associated with digestive tract diseases, including peptic ulcers [37], and ulcerative colitis [38]. The smoking prevalence of Chinese men is substantially higher than that of women [39]; lung function has been already impaired and may not recover within the short term, and may even have a synergistic effect with air pollution, increasing the risk of non-neoplastic digestive system diseases [40]. This may explain the higher estimate of solid fuel effect among males.

Our follow-up subgroup analyses with the data of 2011–2018 also found that people over 65 years exposed to cooking solid fuel had a higher risk of developing digestive problems. A time-series analysis in Nanjing of China provided suggestive evidence that older adults exposed to ambient air pollution are more likely to develop digestive illnesses [14]. Several studies have observed that elderly people are more vulnerable to indoor air pollution damage [41, 42]. Moreover, the elderly in China prefer to use solid living energy than youngers because of its low price [41, 43], increasing exposure to cooking solid fuel. These two reasons may explain why the estimated effect of cooking solid fuel is more pronounced in older groups.

The major strengths of the present study are as follows: the CHARLS adopts a multi-stage stratified probability-proportional-to-size sampling method in both the county/district and village sampling stages. The response rate and data quality of CHARLS rank at the top among similar projects in the world. Professionally trained investigators and staff guarantee the quality of the data collected by CHARLS.

### Limitations

Several limitations should be noted. First, the use of self-reported diagnosis of NNDSD might have underestimated its prevalence, particularly among the older population and those with lower socioeconomic and educational backgrounds. Second, we obtained data on indoor fuel use by questionnaires, not by accurate external and internal exposure measurements. Moreover, we were unable to obtain data on concentrations of environmental air pollution to rule out potential impacts of specific pollutants on the digestive system. However, using measurements of global surface PM2.5 concentrations from the Atmospheric Composition Analysis Group at the University of Washington, we obtained the annual average PM2.5 concentration for each province of China [44]. We conducted baseline analysis of effect of solid fuel use for cooking or/and heating on NNDSD additionally adjusted for PM2.5. The results of the analysis after the inclusion of external confounding are similar to those of the main analysis, which indicates the stability of our results. Future studies might include objective assessment of external pollutants at the individual level. Third, NNDSD in the present study covers all non-neoplastic digestive system diseases, and there is no information about NNDSD subtypes (Peptic ulcer, inflammatory bowel disease, etc.); further studies may explore such associations in different NNDSD subtypes. Finally, the information on heating fuel during follow-up is unavailable. Future studies could incorporate heating fuel usage to fully explore the impact of fuel type conversion on adverse outcomes.

### Conclusion

Indoor solid fuel use for cooking and/or heating was an independent risk factor for non-neoplastic digestive system diseases (NNDSD). Additionally, switching fuel types from solid to clean cooking fuels may help reduce the impact of indoor solid fuel use on NNDSD. The present study highlights the urgency of switching to clean cooking fuels, with major public health implications for developing countries.

## Data Availability

The datasets generated and analyzed during the current study are not publicly available due to separate ongoing original analyses being performed but are available from the corresponding author upon reasonable request.
