# Supplementary material for: Indoor Solid Fuel Use and Non-Neoplastic Digestive System Diseases: A Population-Based Cohort Study Among Chinese Middle-Aged and Older Population
Source: Int J Public Health. 2022 Dec 21;67:1605419. doi: 10.3389/ijph.2022.1605419 (PMC9810631; doi:10.3389/ijph.2022.1605419)
Supplement: Supplementary file 1 [file DataSheet1.docx]

**International Journal of Public Health**

**Indoor solid fuel use and non-neoplastic digestive system diseases: A population-based cohort study among Chinese middle-aged and older population**

**Supplementary materials**

**Table S1.** Baseline characteristics among included and excluded participants (China Health and Retirement Longitudinal Study, China, 2011).

**Table S2.** The association of household fuel conversion and non-neoplasm digestive system diseases among non-smokers (China Health and Retirement Longitudinal Study, China, 2011, 2013, 2015, 2018).

**Table S3.** The associations between household fuel exposure at baseline and non-neoplasm digestive system diseases stratified by comorbidities using Cox models (China Health and Retirement Longitudinal Study, China, 2011, 2013, 2015, 2018).

**Table S4.** The mediating role of lung diseases in the association between solid fuel use and non-neoplasm digestive system diseases (China Health and Retirement Longitudinal Study, China, 2011, 2013, 2015, 2018).

**Table S5.** The associations between baseline household fuel exposure and non-neoplasm digestive system diseases diagnosed during follow-up using Cox models additionally adjusted for the concentration of PM2.5 (China Health and Retirement Longitudinal Study, China, 2011, 2013, 2015, 2018).

**Fig. S1.** Causal diagram showing selection of covariates for confounding control (China Health and Retirement Longitudinal Study, China, 2011, 2013, 2015, 2018).

**Fig. S2.** The subgroup analysis of the association between cooking and heating fuel combined exposure and non-neoplasm digestive system diseases compared with both clean fuel for cooking and heating (China Health and Retirement Longitudinal Study, China, 2011, 2013, 2015, 2018).

| **Table S1.** Baseline characteristics among included and excluded participants (China Health and Retirement Longitudinal Study, China, 2011). | | | |
| --- | --- | --- | --- |
| **Variables** | **Included**  **(N=7884)** | **Included**  **(N=2962)** | **Excluded**  **(N=9799)** |
| **Sex** |  |  |  |
| male | 3842(48.7) | 1397(47.2) | 4616(47.1) |
| female | 4042(51.3) | 1565(52.8) | 5174(52.8) |
| Missing |  |  | 9(0.1) |
| **Age (years)** |  |  |  |
| 45-65 | 6091(77.3) | 2061(69.6) | 6824(69.6) |
| >65 | 1793(22.7) | 901(30.4) | 2975(30.4) |
| **Region** |  |  |  |
| city/town | 7331(93.0) | 2763(93.3) | 8410(85.8) |
| village | 553(7.0) | 199(6.7) | 1362(13.9) |
| Missing |  |  | 27(0.3) |
| **smoking** |  |  |  |
| smokers | 3066(38.9) | 1251(42.2) | 3874(39.5) |
| non-smokers | 4818(61.1) | 1711(57.8) | 5819(59.4) |
| Missing |  |  | 106(1.1) |
| **Marital status** |  |  |  |
| married | 7041(89.3) | 2280(77.0) | 8352(85.2) |
| unmarried | 843(10.7) | 682(23.0) | 1447(14.8) |
| **economy standard** |  |  |  |
| poor | 3351(42.5) | 1304(44.0) | 5344(54.5) |
| average | 4309(54.6) | 1559(52.6) | 4198(42.8) |
| high | 224(2.8) | 99(3.4) | 257(2.7) |
| **Education** |  |  |  |
| illiteracy or informal education | 3535(44.8) | 1423(48.0) | 4314(44.0) |
| elementary school or above | 4349(55.2) | 1539(52.0) | 5485(46.0) |
| **Hypertension** |  |  |  |
| yes | 1738(22.0) | 692(23.4) | 2551(26.0) |
| no | 6146(78.0) | 2270(76.6) | 7158(73.1) |
| Missing |  |  | 90(0.9) |
| **Liver disease** |  |  |  |
| yes | 218(2.8) | 85(2.9) | 458(4.6) |
| no | 7666(97.2) | 2877(97.1) | 9234(94.3) |
| Missing |  |  | 107(1.1) |
| **Lung disease** |  |  |  |
| yes | 625(7.9) | 258(8.7) | 897(9.1) |
| no | 7259(92.1) | 2704(91.3) | 8797(89.8) |
| Missing |  |  | 105(1.1) |
| **Cancer or malignant tumor** |  |  |  |
| yes | 64(0.8) | 20(0.7) | 117(1.19) |
| no | 7820(99.2) | 2942(99.3) | 9572(97.7) |
| Missing |  |  | 110(1.1) |
| **Heart disease** |  |  |  |
| yes | 660(8.4) | 261(8.8) | 875(8.9) |
| no | 7224(91.6) | 2701(91.2) | 8816(90.0) |
| Missing |  |  | 108(1.1) |
| **Stroke** |  |  |  |
| yes | 145(1.8) | 62(2.1) | 269(2.7) |
| no | 7739(98.2) | 2900(97.9) | 9425(96.2) |
| Missing |  |  | 105(1.1) |
| **Kidney disease** |  |  |  |
| yes | 381(4.8) | 149(5.0) | 553(5.6) |
| no | 7503(95.2) | 2813(95.0) | 9133(93.2) |
| Missing |  |  | 113(1.2) |
| Data are presented as n (%) or mean ± standard deviation (SD). | | | |

| **Table S2.** The association of household fuel conversion and non-neoplasm digestive system diseases among non-smokers (China Health and Retirement Longitudinal Study, China, 2011, 2013, 2015, 2018). | | | | | |
| --- | --- | --- | --- | --- | --- |
| **Variables** | **No. of**  **Events** | **Rate ^b^**  **(1/10^3^)** | **HR (95%CI) ^a^** | | **P-value** |
| **Consistent fuel type** | | | | | |
| Always clean fuel | 99 | 18.48 | reference | 1 |  |
| Always solid fuel | 86 | 27.68 | model 1 | 1.18(1.11,1.23) | <0.001 |
|  |  |  | model 2 | 1.15(1.09,1.22) | <0.001 |
|  |  |  | model 3 | 1.13(1.09,1.24) | 0.004 |
| **Had switched fuel type** | | | | | |
| Always solid fuel | 86 | 31.68 | reference | 1 |  |
| Solid to clean fuel | 41 | 22.24 | model 1 | 0.67(0.47,0.90) | 0.001 |
|  |  |  | model 2 | 0.65(0.48,0.92) | 0.002 |
|  |  |  | model 3 | 0.65(0.47,0.94) | 0.001 |
| ^a^ Model 1 is a crude model; Model 2 adjusts for age, gender, and education level; Model 3 adjusts for gender, age, education level, residence, economy level, and marital status.  ^b^ Incidence rate per 1000 person-years of follow-up, equal to (number of non-neoplasm digestive system diseases events)/(person-years) *1000. | | | | | |

| **Table S3.** The associations between household fuel exposure and non-neoplasm digestive system diseases diagnosed at baseline using stratified by comorbidities using Cox models (China Health and Retirement Longitudinal Study, China, 2011, 2013, 2015, 2018). | | | | | | |
| --- | --- | --- | --- | --- | --- | --- |
| **Variables** | **Cooking fuel use** | | | **Heating fuel use** | | |
|  | **Events (N)** | **Rate (1/10^3^)** | **aHR (95%CI)** | **Events (N)** | **Rate (1/10^3^)** | **aHR (95%CI)** |
| **No hypertension** | 396 | 18.66 | 1(reference) | 391 | 18.76 | 1(reference) |
|  | 436 | 22.85 | 1.21(1.05, 1.39) | 442 | 22.71 | 1.19(1.04, 1.37) |
| **Hypertension** | 114 | 18.08 | 1 | 119 | 18.20 | 1 |
|  | 115 | 22.50 | 1.22(0.94, 1.58) | 110 | 22.55 | 1.21(0.93, 1.58) |
| **No liver diseases** | 488 | 18.23 | 1 | 488 | 18.31 | 1 |
|  | 531 | 22.53 | 1.21(1.07, 1.38) | 531 | 22.42 | 1.20(1.06, 1.36) |
| **Liver diseases** | 22 | 29.02 | 1 | 22 | 30.10 | 1 |
|  | 20 | 31.90 | 1.10(0.59, 2.03) | 20 | 30.58 | 1.04(0.56, 1.91) |
| **No lung diseases** | 462 | 18.03 | 1 | 460 | 18.09 | 1 |
|  | 479 | 21.68 | 1.19(1.05, 1.36) | 481 | 21.59 | 1.18(1.03, 1.34) |
| **Lung diseases** | 48 | 25.20 | 1 | 50 | 25.63 | 1 |
|  | 72 | 34.22 | 1.30(0.90, 1.89) | 70 | 34.01 | 1.27(0.87, 1.84) |
| **No cancer or malignant tumor** | 498 | 18.25 | 1 | 495 | 18.23 | 1 |
|  | 549 | 22.84 | 1.23(1.08, 1.40) | 548 | 22.67 | 1.22(1.08, 1.38) |
| **Cancer or malignant tumor** | 12 | 49.38 | 1 | 11 | 47.01 | 1 |
|  | 2 | 12.35 | 0.18(0.04, 1.03) | 3 | 25.64 | 0.28(0.07, 1.13) |
| **No heart diseases** | 447 | 17.70 | 1 | 431 | 17.35 | 1 |
|  | 474 | 21.24 | 1.17(1.02, 1,33) | 490 | 21.55 | 1.21(1.06, 1.38) |
| **Heart diseases** | 63 | 27.69 | 1 | 79 | 31.00 | 1 |
|  | 77 | 40.98 | 1.50(1.07, 2.10) | 61 | 37.98 | 1.23(0.88, 1.73) |
| **No stroke** | 495 | 18.31 | 1 | 493 | 18.35 | 1 |
|  | 536 | 22.54 | 1.21(1.07 1.38) | 538 | 22.46 | 1.20(1.06, 1.36) |
| **Stroke** | 15 | 30.36 | 1 | 17 | 32.76 | 1 |
|  | 15 | 36.32 | 1.20(0.58, 2.51) | 13 | 33.51 | 1.02(0.48, 2.19) |
| **No kidney diseases** | 479 | 18.19 | 1 | 483 | 18.44 | 1 |
|  | 510 | 22.21 | 1.20(1.15, 1.36) | 506 | 21.91 | 1.16(1.03, 1.32) |
| **Kidney diseases** | 31 | 25.83 | 1 | 27 | 22.69 | 1 |
|  | 41 | 33.31 | 1.34(0.83, 2.16) | 45 | 36.26 | 1.71(1.05, 2.77) |
| **Number of comorbidities** |  |  |  |  |  |  |
| **0** | 300 | 17.01 | 1 | 294 | 17.16 | 1 |
|  | 316 | 20.53 | 1.18(1.01, 1.39) | 322 | 20.26 | 1.16(0.98, 1.36) |
| **1** | 132 | 18.27 | 1 | 132 | 17.60 | 1 |
|  | 151 | 22.89 | 1.23(0.57, 1.96) | 151 | 23.90 | 1.34(1.06, 1.71) |
| **≥2** | 78 | 29.21 | 1 | 84 | 30.52 | 1 |
|  | 84 | 38.08 | 1.32(0.97, 1.80) | 78 | 36.90 | 1.21(0.89, 1.66) |
| Model adjusts for age, sex, educational level, marital status, residence region, and smoking status. Rate: incidence rate per 1000 person-years of follow-up, equal to (number of non-neoplasm digestive system diseases events)/(person-years)$\times$1000. | | | | | | |

| **Table S4.** The mediating role of lung diseases in the association between solid fuel use and non-neoplasm digestive system diseases (China Health and Retirement Longitudinal Study, China, 2011, 2013, 2015, 2018). | | | | |
| --- | --- | --- | --- | --- |
| Outcome | HR_TE_* | HR_CDE_† | HR_PE_† | Proportion eliminated (%) † |
| Baseline cooking fuel type | | | | |
| NNDSD | 1.21(1.07, 1.36) | 1.18(1.05, 1.35) | 1.03(1.01, 1.15) | 14.3(3.2, 25.6) |
| Baseline heating fuel type | | | | |
| NNDSD | 1.20(1.06, 1.35) | 1.17(1.03, 1.34) | 1.02(1.00, 1.16) | 15.0(0, 27.3) |
| Data are presented with the 95% CI. *Controlled for age, sex, educational level, marital status, residence region, smoking status. † Controlled for lung diseases, age, sex, educational level, marital status, residence region, smoking status. HR_PE_ = (HR_TE_/HR_CDE_). Proportion eliminated = (HR_TE_$-$HR_CDE_)/ (HR_TE_$-$1), only present if the direction of CDE and PE was the same. The bootstrapped CIs for HR_PE_ and proportion eliminated were obtained using 100 replicates. | | | | |

| **Table S5.** The associations between baseline household fuel exposure and non-neoplasm digestive system diseases diagnosed during follow-up using Cox models additionally adjusted for the concentration of PM2.5 (China Health and Retirement Longitudinal Study, China, 2011, 2013, 2015, 2018). | | | |
| --- | --- | --- | --- |
| **Variables** |  |  |  |
|  | **Events (Rate (1/10^3^))** |  | **aHR (95%CI)** |
| **Cooking fuel** |  |  |  |
| Clean | 510(18.62) | Reference | 1 |
| Solid | 551(22.64) | Model 1 | 1.21(1.07, 1.36) |
|  |  | Model 2 | 1.19(1.05, 1.35) |
| **Heating fuel** |  |  |  |
| Clean | 510(18.53) | Reference | 1 |
| Solid | 551(22.77) | Model 1 | 1.19(1.05, 1.35) |
|  |  | Model 2 | 1.17(1.03, 1.33) |
| **Cooking and heating fuel** |  |  |  |
| Both clean for cooking and heating | 362(17.97) | Reference | 1 |
| Clean for cooking and solid for heating | 148(20.04) | Model 1 | 1.10(0.91, 1.33) |
|  |  | Model 2 | 1.08(0.89, 1.31) |
| Clean for heating and solid for cooking | 148(20.44) | Model 1 | 1.12(0.93, 1.36) |
|  |  | Model 2 | 1.11(0.92, 1.35) |
| Both solid for cooking and heating | 403(23.77) | Model 1 | 1.29(1.12, 1.50) |
|  |  | Model 2 | 1.26(1.09, 1.47) |
| Model 1 adjusts for age, sex, educational level, marital status, residence region, smoking status, and PM2.5 concentration calculated by population weighting. Model 2 adjusts for age, sex, educational level, marital status, residence region, smoking status, and PM2.5 concentration calculated by geographical weighting. Rate: incidence rate per 1000 person-years of follow-up, equal to (number of non-neoplasm digestive system diseases events)/(person-years)$\times$1000. | | | |

**
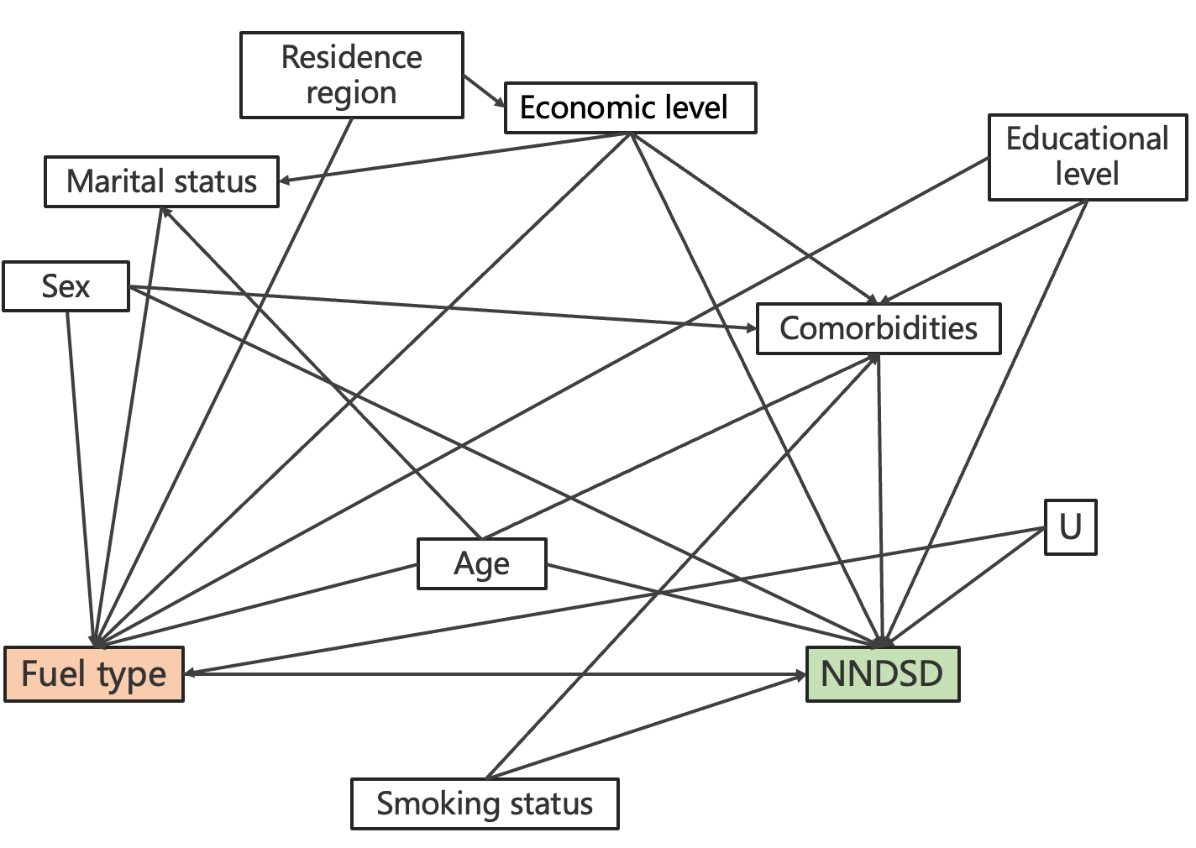
**

**Fig. S1.** Causal diagram showing selection of covariates for confounding control (China Health and Retirement Longitudinal Study, China, 2011, 2013, 2015, 2018).

Comorbidities: hypertension comorbidity, diabetes, dyslipidemia, cardiovascular disease, stroke, liver diseases, lung diseases, cancer or malignant tumor, and kidney diseases; U: unmeasured variables.


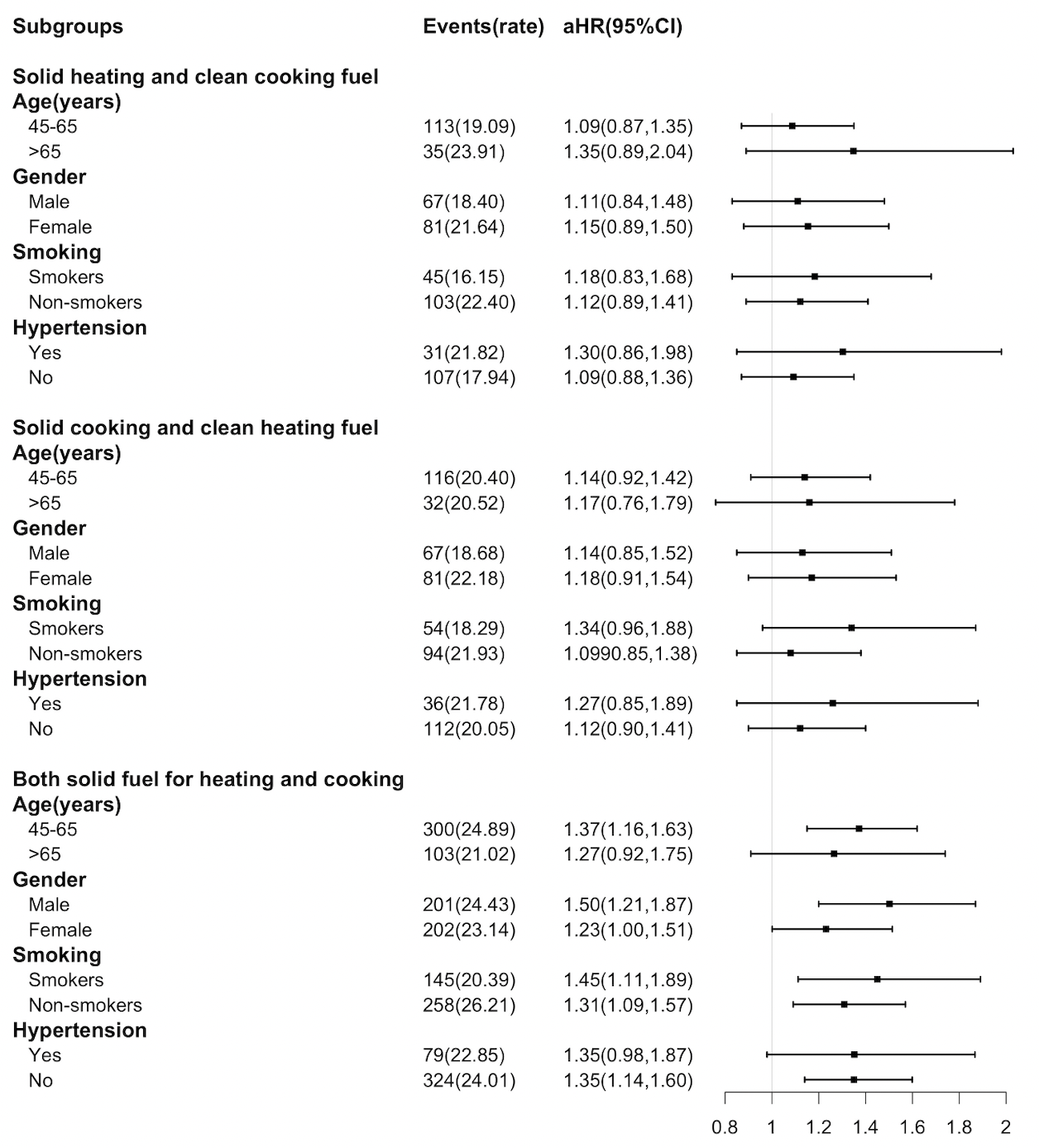


**Fig. S2.** The subgroup analysis of the association between cooking and heating fuel combined exposure and non-neoplasm digestive system diseases compared with both clean fuel for cooking and heating (China Health and Retirement Longitudinal Study, China, 2011, 2013, 2015, 2018).

The effects are presented by aHR with 95% CI. The reference group is clean fuel for both heating and cooking. The multivariable-adjusted model is the fully adjusted model 3 described above. Rate: incidence rate per 1000 person-years of follow-up, equal to (number of non-neoplasm digestive system diseases events)/(person-years) *1000.
